# Atmospheric pressure air microplasma current time series for true random bit generation

**DOI:** 10.1038/s41598-020-77956-5

**Published:** 2020-12-01

**Authors:** Anis Allagui, Sohaib Majzoub, Ahmed S. Elwakil, Andrea Espinel Rojas, Hussain Alawadhi

**Affiliations:** 1 Department of Sustainable and Renewable Energy Engineering, University of Sharjah, PO Box 27272, Sharjah, United Arab Emirates; 2 Center for Advanced Materials Research, Research Institute of Sciences and Engineering, University of Sharjah, PO Box 27272, Sharjah, United Arab Emirates; 3 Department of Mechanical and Materials Engineering, Florida International University, Miami, FL 33174 United States; 4 Department of Electrical Engineering, University of Sharjah, PO Box 27272 Sharjah, United Arab Emirates; 5 Nanoelectronics Integrated Systems Center, Nile University, Cairo, 12588 Egypt; 6 Department of Electrical and Computer Engineering, University of Calgary, Calgary, AB T2N 1N4 Canada; 7 Department of Applied Physics and Astronomy, University of Sharjah, PO Box 27272, Sharjah, United Arab Emirates

**Keywords:** Electrical and electronic engineering, Plasma physics

## Abstract

Generating true random bits of high quality at high data rates is usually viewed as a challenging task. To do so, physical sources of entropy with wide bandwidth are required which are able to provide truly random bits and not pseudorandom bits, as it is the case with deterministic algorithms and chaotic systems. In this work we demonstrate a reliable high-speed true random bit generator (TRBG) device based on the unpredictable electrical current time series of atmospheric pressure air microplasma (APAMP). After binarization of the sampled current time series, no further post-processing was needed in order for the bitstreams to pass all 15 tests of the NIST SP 800-22 statistical test suite. Several configurations of the system have been successfully tested at different sampling rates up to 100 MS/s, and with different inter-electrode distances giving visible/non-visible optical emissions. The cost-effectiveness, simplicity and ease of implementation of the proposed APAMP system compared to others makes it a very promising solution for portable TRBGs.

## Introduction

Microplasma confined to dimensions in the order of or below the millimeter are known to be remarkably stable at high pressures. This allows self-sustained and continuous operation without filamentation and glow-to-arc transition^1^. In addition, a complete microplasma system can be made light weight and small in size in different design geometries and configurations^2–5^. When air (at atmospheric pressure) is used as the plasma gas, and because no special housing or vacuum equipment are needed, the microplasma system becomes cost-effective and easy to operate^6^. These advantages make microplasmas ideal for portable systems and instruments for chemical^7^ and spectrochemical^8–10^ analysis, thin film deposition^11^, NO_x_ and SO_x_ remediation and treatment of volatile organic compounds^12^, biomedical decontamination and dental sterilization^2,13–15^, and many other applications^16,17^.

Most, if not all, of these applications rely on the fact that microplasmas provide a rich environment of high-energy electrons and other reactive, excited and metastable species, ultraviolet radiation, and intense electric fields without the generation of excessive heat^2^. In high-pressure and atmospheric pressure systems in particular, charged and uncharged species are actually in non-local equilibrium with the electric field due to the large and non-monotonous profiles of the latter, and also due to the small dimensions of the system^2^. This non-equilibrium character of microplasma and erratic movement of its elemental species which manifests itself as high-frequency electrical current fluctuations (coupled with others, such as acoustic and optical fluctuations^18^) has been shown to be useful for another type of application: high-rate random bit generation (RBG)^19^. RBGs are very important for cryptographic systems, secure communication, Monte Carlo numerical computations, statistical research, randomized algorithms, etc. The microplasma system we have investigated before^19^ was submerged in an electrolyte. The glow discharge is generated between the tip of a needle electrode surrounded by a gaseous sheath and a concentrated anolyte or catholyte using low dc voltages (< 100 Vdc)^20–23^. From the dynamic analysis of its current time series in terms of phase-space portrait, fractal dimension, largest Lyapunov exponent and power spectra, we established that the electrochemical plasma undergoes a transition from quasi-periodic to chaotic and quasi-hyper-chaotic behavior as the applied voltage is increased^21^. We also showed that the binary sequences generated from the current time signals obtained at large voltages unambiguously pass (after simple post-processing) all 15 tests of NIST SP 800-22 Statistical Test Suite^19,24^. However, despite these promising results, the fact that liquids and evaporated corrosive gases were involved in the microplasma process, posed limitations on their portability, packaging and ease-of-maintenance.

To overcome some of these limitation, in this paper we rely on the electrical current fluctuations in atmospheric pressure air microplasma (APAMP) as a source of entropy for RBG. The system is described in the “Experimental” section, and it comprises a high-bandwidth current probe for time-resolved measurements of current intensity, analog-to-digital conversion, and optionally applying a simple binarization procedure on the raw data. The microplasma circuit consists of off-the-shelf standard electrical components, which makes it simple and cost-effective when compared to photonic and optical signals-based RBGs^25–30^. The APAMP system has a relatively high throughput rate (bitstreams of up to 100 Mbit/s that pass all 15 NIST SP 800-22 tests without applying any digital post-processing routines, “Results” section), and is resistant to external attacks given the high-voltage requirements for the microplamsa. A comparison with other existing RBG systems and processes is provided and discussed in the “Discussion” section.

## Experimental

**Figure 1:**
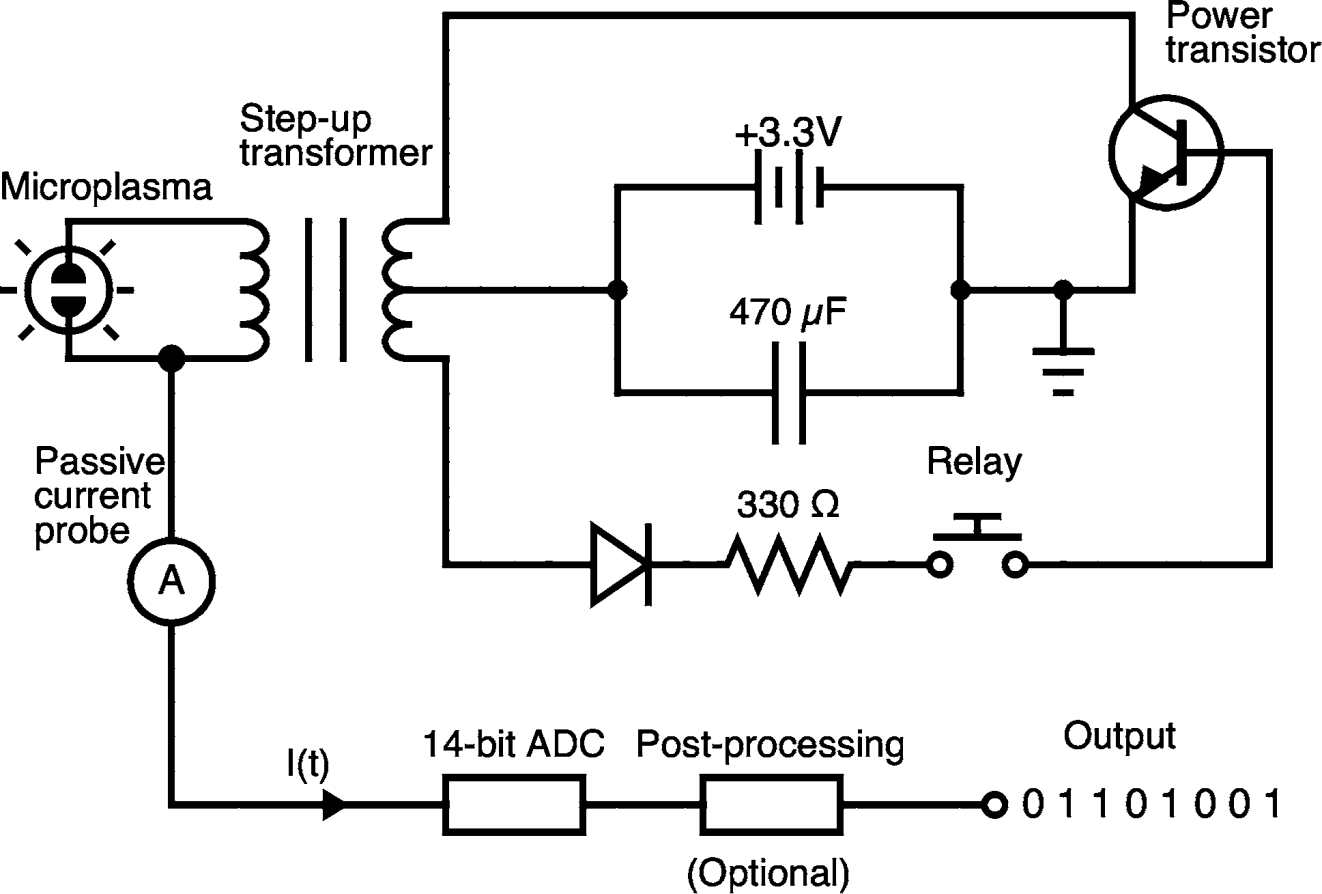
Circuit diagram of a battery-powered atmospheric pressure air microplasma (APAMP) system used for random bit generation (ADC: analog-to-digital converter).

Figure 1 depicts a circuit diagram of the APAMP circuit designed and investigated in this study for RBG. A photograph of the prototype is provided in Fig. S1a. The circuit consists of a high-power transistor, a center-tap step-up flyback transformer and a diode powered by a 3.3 V, 4400 mA h rechargeable lithium-ion battery (LIB). An arc discharge is ignited and sustained in free air between two needle-like electrodes (primary side of the transformer) of 1 mm in diameter aligned facing each other at a distance of a few millimeters. The arc current is measured using a high-frequency, high-sensitivity Tektronix CT2 current probe (1.2 kHz to 200 MHz bandwidth at a sensitivity of 1 mA/mV into 50 $\Omega$) connected via a P6041 BNC probe cable to a Digilent Analog Discovery 2 (AD2) data acquisition board (up to 100 MS/s sampling rate, configured in the input voltage range − 2.5 to 2.5 V). The board is connected via USB 2.0 to a PC for saving and analyzing the collected data, and for controlling the relay (Fig. 1, Fig. S1). The maximum capacity of AD2’s internal buffer is 16384 ($=2^{14}$) samples at a time. To acquire longer bitstreams needed for applying the NIST SP 800-22 tests (considered to be the de facto standard statistical test suite for randomness studies for RBG applications) and other batteries of tests, a C-script was used to read and save the 16384 samples iteratively to accumulate the target number of samples while the microplasma circuit remained continuously ON (see flowchart in Fig. S2). We tested four different prototypes of the same circuit (cost does not exceed USD 50 per prototype, excluding the current probe and data acquisition board), and the results given here are those obtained from one of them and typical for all (see Fig. S1). More than 120 Gbit of data were collected over several weeks of testing.

## Results

### Raw electrical current time series

Upon the application of a high enough dc voltage, a visible current channel or arc is established between the cathodic and anodic tips of the system through electron thermionic emission or field emission or both from the cathode^31^. Positive ions are then accelerated in the opposite direction to strike the cathode, and thereby transferring their energy to it, which allows the maintenance of sufficient temperature to keep up the thermal emission of electrons^32^.

**Figure 2:**
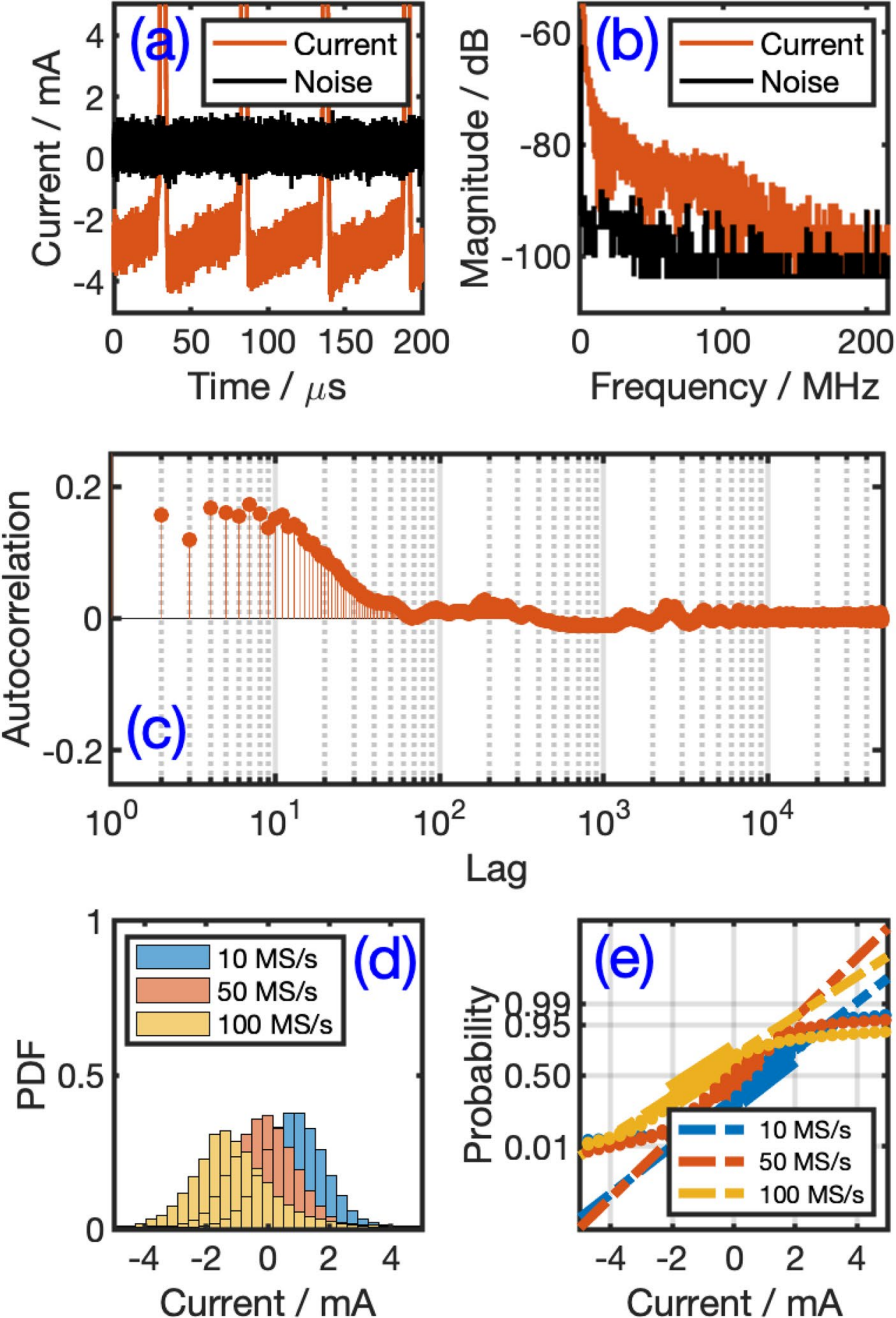
Analysis of current signal collected from the atmospheric pressure air microplasma (APAMP) system: (**a**) Typical sample of current time series at a rate of 10 MS/s along with the noise floor, (**b**) Fast Fourier transform (FFT) of the current and noise signals obtained with a digital Tektronix TBS2104 oscilloscope (sampled at 500 MS/s), (**c**) Autocorrelation plot of a sample of current time series, (**d**) Probability distribution functions (PDF) estimate of current time series samples of 100 kpts in size collected at the rates of 10, 50 and 100 MS/s (the height of each bar is the relative number of observations), (3) Normal probability plots of the samples in (**c**) (in dots) aligned with the theoretical normal distribution (dashed lines); the solid thick lines connect the first and third quartiles of the data.

**Figure 3:**
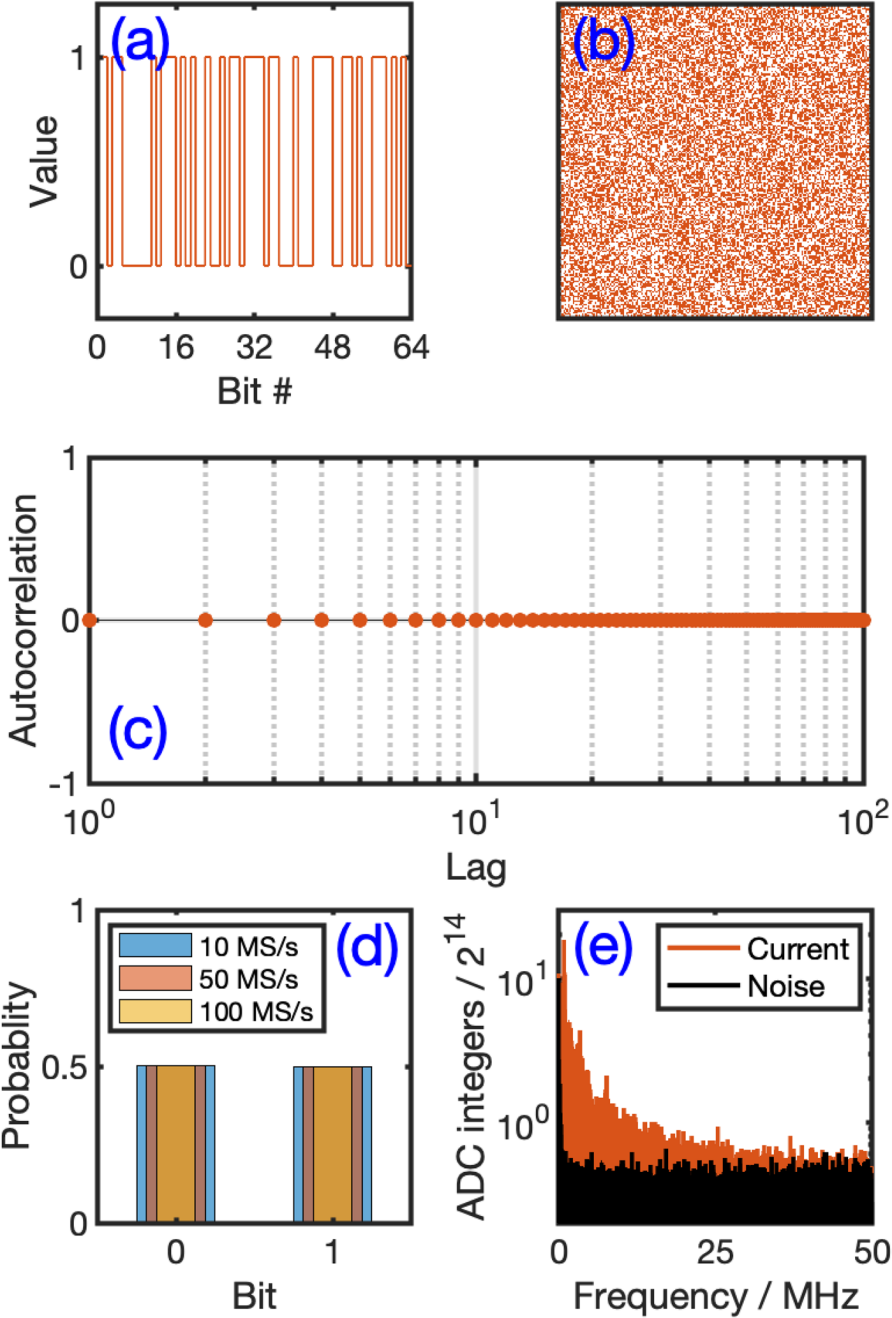
Analysis of bitstream obtained from the atmospheric pressure air microplasma (APAMP) current signal: (**a**) Bit values of a typical sequence of 64 successive bits in a 1D stair plot. (**b**) 2D raster image of randomly selected $200\times 200$ consecutive bits which does not show, at least visually, any particular concentration of pockets or patterns of zeros or ones, (**c**) Autocorrelation plot of a sample of binary data collected at 100 MS/s (correlation is positive and anti-correlation is negative), (**d**) Histograms of 24576000-long bitstreams generated from current time series collected at 10, 50 and 100 MS/s, (**e**) FFT of data collected directly from the 14-bit ADC register of the DAQ board (current and noise) at 100 MS/s.

A typical 200 μs sample of current time series ($I_1, I_2, \ldots , I_n$ at $t_1, t_2, \ldots , t_n$) collected with a time resolution of 2 ns from the APAMP system is shown in Fig. 2a along with the system’s background noise. The signal exhibits alternating current spikes and constrictions of different durations (i.e. different frequencies) and relatively low intensities superseding each wave of high spikes (in the order of a few amperes) corresponding to the transformer charging/discharges responses. This type of sustained and erratic behavior is typical and consistently observed irrespective of the electrodes’ orientations (i.e. horizontal, vertical, or in between) or the prototype we tested as long as a critical distance of a few millimeters between the two is maintained. An increase of this distance weakens the intensity of the plasma arc current. In Fig. 2b, we show the fast Fourier transform (FFT) of the current signal and the noise floor (obtained with a digital Tektronix TBS2104 oscilloscope with sampling at 500 MS/s), and a screenshot of their power spectra (measured with a real-time Tektronix 2711 spectrum analyzer of bandwidth 9 kHz–1.8 GHz) is provided in Fig. S3a. The power of the current signal is distributed over a wide frequency band with a slope of approximately − 0.35 dB/MHz from 10 to 50 MHz, and then with a slope of − 0.06 dB/MHz from 50 to 190 MHz. Above this limiting frequency the power of the signal fades out to reach that of the noise level. An estimate of the (normalized) autocorrelation:

1 $$\begin{aligned} R[j]= \frac{\sum _{i=1}^{n-j} I(i)I(i+j) }{n-j} \end{aligned}$$

where *n* is the length of the sequence, is performed on a sample from the current signal and is given in Fig. 2c. The figure compares the original signal with its shifted versions by up to 50000 points (data points are equispaced with $\Delta t= 0.5\,\upmu \text{s}$). One can observe that there is a significant correlation that extends to the level of about 100 points and thus there are some memory effects in the system, but then it fades out and remains centered around zero for larger lags. This memory effect will be eliminated after binarization of the data (see Fig. 3c).

We also analyzed the statistical distributions of the raw data collected at different sampling rates (10, 50 and 100 MS/s which are within the bandwidth of the system). In Fig. 2d we show the probability density function (PDF) estimates in histogram forms and in Fig. 2e we show the normal probability plots of the three data samples. Each sample consists of 100 kpts arbitrarily selected from a stream of 16 Mpts. Figure 2e shows that the data series are nicely aligned with the theoretical normal distribution $N(\mu ,\sigma ^2)$ with negligible distortions or asymmetry. We found, for instance, with a confidence interval of 95%, the normal distribution parameter estimates $\hat{\mu } = 0.938 \, [0.934, 0.942]$ mA and $\hat{\sigma } = 2.112 \, [2.109, 2.115]$ mA for the sample collected at 10 MS/s.

### Binary data

The time and frequency-domain analysis and preliminary statical results obtained from the raw current data collected from the APAMP system suggest their potential use as a source of entropy for RBG. To this end, the data have been binarized for further analysis for RBG from NIST SP 800-22 point of view. We followed a procedure similar to the one we reported in Ref.^5,19^ in which we first brought the raw data to be centered around the zero-mean by applying a moving average function and removing the dc shift from the signal. Then, a base-2 representation of the absolute value of the sequence (after scaling up by 10^5^) is generated using the MATLAB function *dec2bin*. Finally, the binary sequence is constructed using the least significant bit (LSB) of each data point.

**Table 1:** Typical results of NIST tests for bitstreams generated from the microplasma current time series for the two cases of visible and non-visible electrical current arc.

Statistical test	Visible electrical current arc									Non-visible electrical current arc		
	10 Mbit/s			50 Mbit/s			100 Mbit/s			100 Mbit/s		
	P-value	Proportion	Assessment	P-value	Proportion	Assessment	P-value	Proportion	Assessment	P-value	Proportion	Assessment
F	0.739918	50/50	Success	0.137282	50/50	Success	0.739918	49/50	Success	0.574903	98/100	Success
BF	0.779188	50/50	Success	0.911413	49/50	Success	0.122325	50/50	Success	0.816537	99/100	Success
CS	0.419021	50/50	Success	0.213309	50/50	Success	0.534146	49/50	Success	0.401199	98/100	Success
R	0.262249	49/50	Success	0.779188	50/50	Success	0.023545	49/50	Success	0.055361	99/100	Success
LR	0.383827	50/50	Success	0.236810	47/50	Success	0.935716	50/50	Success	0.999438	99/100	Success
Rk	0.383827	50/50	Success	0.455937	50/50	Success	0.816537	50/50	Success	0.834308	100/100	Success
FFT	0.419021	50/50	Success	0.122325	48/50	Success	0.657933	49/50	Success	0.236810	100/100	Success
NOT	0.383827	47/50	Success	0.419021	50/50	Success	0.616305	48/50	Success	0.897763	99/100	Success
OT	0.935716	49/50	Success	0.739918	49/50	Success	0.191687	50/50	Success	0.616305	100/100	Success
U	0.739918	50/50	Success	0.574903	50/50	Success	0.419021	50/50	Success	0.213309	10/10	Success
AE	0.883171	49/50	Success	0.066882	49/50	Success	0.289667	50/50	Success	0.224821	100/100	Success
RE	0.002971	15/15	Success	0.834308	26/26	Success	0.911413	22/22	Success	0.012650	15/15	Success
REV	0.437274	15/15	Success	0.012650	26/26	Success	0.122325	22/22	Success	0.275709	15/15	success
S	0.262249	49/50	Success	0.657933	49/50	Success	0.779188	48/20	Success	0.935716	99/100	Success
LC	0.236810	49/50	Success	0.383827	47/50	Success	0.699313	50/50	Success	0.616305	100/100	Success

When arcing is visible (inter-electrode distance of $\sim$ 1 mm), we tested bitstream of 24 Mbit in length (50 sequences of 480,000 bits) collected at the sampling rates of 10 MS/s, 50 MS/s and 100 MS/s. For the case of non-visible arcing (inter-electrode distance of $\sim$ 3–4 mm), we tested bitstream of 16 Mbit in length (100 sequences of 160,000 bits, for Universal test 10 sequences of 1,600,000 bits) collected at the sampling rate of 100 MS/s. (F stands for test Frequency, BF for Block Frequency, CS for Cumulative Sums, R for Runs, LR for Longest Run, Rk for Rank, FFT for Fast Fourier Transform, NOT for Non Overlapping Template, OT for Overlapping Template, U for Universal, AE for Approximate Entropy, RE for Random Excursions, REV for Random Excursions Variant, S for Serial, and LC for Linear Complexity).

Some statistical information on the binarized data are given in Fig. 3 (see details in the figure caption). Figure 3a shows a stair plot of 64 successive bits, and Fig. 3b illustrates a 2D raster image of 40,000 consecutive bits that indicates, at least visually, that there are no obvious patterns or structures in the binary data. Figure 3d shows a uniform distribution for the bits “0” and “1”, i.e. probability of occurrence $P(0)\approx 0.4999, 0.5002, 0.4999$ for the rates 10, 50 and 100 MS/s, respectively. We also computed the number of times the bit “0” is generated knowing that the previous one was a “0” (denoted “00”) and did the same for “01”, “10” and “11” (i.e., conditional probability *p*(*x*|*y*)). We found in a sample size of 24576000 bits generated from the current time series collected at the rate of 10 MS/s the respective times of occurrences of 6148108, 6142061, 6142062 and 6143768. These values correspond to the probabilities 0.2502, 0.2499, 0.2499 and 0.2500 for “00”, “01”, “10” and “11”, respectively as would be expected for random bit series. This means that there is no particular preference to any of them and thus no form of memory of at least the prior state during the bit generation process^29^. Higher-order correlation could be established from auto-correlation analysis as shown in Fig. 3c for up to 100 bits of shift. Similar results were found for all tested sampling rates (not shown here). For the autocorrelation test we converted the bit sequence $X_1, X_2, \ldots , X_n$ of “0”s and “1”s into another sequence $Y_1, Y_2, \ldots , Y_n$ of “+ 1”s and “− 1”s via $Y_i = 2 X_i -1$ so that the correlation will be positive and the anti-correlation will be negative^33^. Contrary to the results obtained from the raw current data, the autocorrelation coefficients of the binary data are practically zero for any number of shifted bits, and thus no memory effects remain. Finally, in Fig. 3d we show the power spectra of sampled (at 100 MS/s) current time series obtained directly from the 14-bit resolution analog-to-digital converter (ADC) register of the AD2 board along with the sampled noise. The graph clearly shows that the sampled data, even with superposed noise contributions from the AD2 board, are above the noise floor.

In Tables 1 we present the statistical results (P-value and proportion of sequences that passed the test) of the 15 NIST SP 800-22 tests obtained under the different conditions, while recognizing that passing these tests does not rigorously guarantee the randomness of the bitstreams. For the execution of the NIST randomness tests, we used the following parameters (unless mentioned otherwise): (i) $\alpha =0.01$ (significance level), (ii) block length for (a) Block Frequency Test is $M=128$, (b) NonOverlapping Template Test is $m=9$, (c) Overlapping Template Test is $m=9$, (d) Approximate Entropy Test is $m= 10$, (e) Serial Test is $m=16$, (f) Linear Complexity Test is $M=500$. The table shows the results computed from 24 Mbits obtained from binarized current signals collected at three sampling rates, 10, 50 and 100 MS/s. The time taken to collect all 24 Mbits was just 3.3 s, in addition to approximately 2.0 s needed for the relay to switch the plasma ON and get it stabilized. The P-value defined as “*the probability that a perfect random number generator would have produced a sequence less random than the tested sequence*”^24^ and associated with each test, is larger than $\alpha =0.01$ for all tests and for all sampling rates. In the table, this is indicated by “success” under the columns “Assessement”. If the P-value is less than $\alpha$, then the null hypothesis $H_0$ that the sequence is truly random is rejected, and therefore it is not considered to be random, also from the point of view of the specific test. The proportion of sequences that passed the tests for the values of P-value should be greater than $\tilde{p}-3 \sqrt{\tilde{p}(1-\tilde{p})/m}$, where $\tilde{p}=1-\alpha$ and *m* is the sample size. For our case where $m=50$ (most of the tests in Table 1) and $\alpha =0.01$, the proportion should lie above 0.947786, which means a minimum pass rate of approximately 47/50 binary sequences. The NIST SP 800-90B package was used to estimate the min-entropy of the data coming from the RNG device, which ideally would be 1 Shannon per bit^34^. We found a min-entropy:

2 $$\begin{aligned} H=\min \limits _{1\le i \le n} (-\ln p_i) = 0.995725 \end{aligned}$$

for the binarized current time series data collected at 10 MS/s. The data were verified to pass the i.i.d. (independent and identically distributed) and Restart tests. The min-entropy was found similarly high and very close to 1 Shannon per bit for the data collected at 50 and 100 MS/s, i.e. 0.994468 and 0.994888, respectively (see Figs. S5, S6 and S7).

We were also interested to see how the RBG can be affected if the inter-electrode distance is increased. We tested the scenario in which we pulled apart the two electrodes to a distance of $\sim$ 3–4 mm. With this configuration, an acoustic signal emanating from the microplasma environment can be heard but no visible optical emissions can be observed with the naked eye. In spite of that, the resulting current time series still appears to be intermittent and disorganized, as shown in Fig. S4. In addition, Fig. S3b shows the power spectra of the signal which is still above the noise floor. The NIST SP 800-22 tests conducted on the binarized data following the same procedure aforementioned were all passed, as shown in Table 1 (last three columns) for the sampling rate of 100 MS/s. The min-entropy estimate was found to be 0.995938 (Fig. S8). This demonstrates that the inter-electrode distance has, to a certain extent, little effect on the RBG performance of our APAMP system. In addition, because the high current spikes observed when visible arc plasma was in place are considerably reduced, the degradative effects of electrodes over-heating is also reduced.

**Table 2:** Typical NIST SP 800-22 tests results performed on 100 Mbit-long bitstreams collected from Bits 3 to 6 from the ADC register at the sampling rate of 100 MS/s.

Statistical test	Bit 3			Bit 4			Bit 5			Bit 6		
	P-value	Proportion	Assessment	P-value	Proportion	Assessment	P-value	Proportion	Assessment	P-value	Proportion	Assessment
F	0.1088	98/100	Success	0.2493	98/100	Success	0	0/100	Fail	0	0/100	Fail
BF	0.9114	98/100	Success	0.5544	98/100	Success	0	0/100	Fail	0	0/100	Fail
CS	0.4823	98/100	Success	0.4466	97/100	Success	0	0/100	Fail	0	0/100	Fail
R	0.4373	99/100	Success	0.8832	99/100	Success	0	0/100	Fail	0	0/100	Fail
LR	0.4190	99/100	Success	0.2622	98/100	Success	0	0/100	Fail	0	0/100	Fail
Rk	0.0856	99/100	Success	0.6787	99/100	Success	0.6371	100/100	Success	0.2368	100/100	Success
FFT	0.8343	100/100	Success	0.4012	99/100	Success	0.2493	99/100	Success	0	0/100	Fail
NOT	0.4718	98/100	Success	0.5151	98/100	Success	0.3116	99/100	Success	0.2696	98/100	Success
OT	0.3505	100/100	Success	0.7598	99/100	Success	0.0010	95/100	Fail	0	0/100	Fail
U	0.4944	97/100	Success	0.1816	99/100	Success	0	0/100	Fail	0	0/100	Fail
AE	0.6371	98/100	Success	0.6163	100/100	Success	0	0/100	Fail	0	0/100	Fail
RE	0.5689	51/52	Success	0.0533	16.8/17	Success	0	0/100	Fail	0	0/100	Fail
REV	0.4292	51/52	Success	0.0609	17/17	Success	0	0/100	Fail	0	0/100	Fail
S	0.5422	99/100	Success	0.8069	100/100	Success	0.0966	97/100	Success	0	0/100	Fail
LC	0.5141	99/100	Success	0.3041	100/100	Success	0.4559	99/100	Success	0.7598	97/100	Success

The inter-electrode distance of the APAMP system is $\sim$ 3–4 mm (no visible current arc). (F stands for test Frequency, BF for Block Frequency, CS for Cumulative Sums, R for Runs, LR for Longest Run, Rk for Rank, FFT for Fast Fourier Transform, NOT for Non Overlapping Template, OT for Overlapping Template, U for Universal, AE for Approximate Entropy, RE for Random Excursions, REV for Random Excursions Variant, S for Serial, and LC for Linear Complexity).

Finally, we have tested the individual bitstreams directly acquired from the ADC register of the AD2 board. With this, the binarization of the raw current time series data is bypassed, which in turn makes the overall TRBG process faster. The results reported here are those for the no visible arcing condition, but similar results were obtained when arcing is visible. In Table 2, we present the NIST SP 800-22 statistical analysis performed for the data of Bits 3, 4, 5 and 6, without any post-processing work. Data from Bits 1 and 2 were discarded because they are close to the noise floor of the system, data from Bits 3 and 4 passed all 15 NIST SP 800-22 tests, whereas data from Bits 5 and 6 failed several tests. In Table S1, we also show the NIST SP 800-22 tests results for data from Bit 3 (as an example) with the significance level $\alpha =0.001$ instead of 0.01 attesting to the quality of the bitstreams for RBG applications. Further confirmation results with the same dataset are shown in Table S2 for the Dieharder (v. 3.31.1) tests. Results from NIST SP 800-90B package provided a min-entropy estimate of $H= 0.995938$ (Fig. S9). To test any possible mutual relationship between data from Bit 3 and Bit 4, we calculated the coefficient of correlation $C\in [-1,1]$ as follows^35^:

3 $$\begin{aligned} C=\frac{S_{11} S_{00}-S_{10}S_{01}}{\sqrt{ (S_{10}+S_{11}) (S_{01}+S_{00}) (S_{11}+S_{01}) (S_{00}+S_{10}) }} \end{aligned}$$

where $S_{mn}$ with $(m,n)\in \{0,1\}$ represents the number of occurrences of matches with *m* in sequence $(B3)_1, (B3)_2, \ldots , (B3)_n$ (from Bit 3) and *n* in the sequence $(B4)_1, (B4)_2, \ldots , (B4)_n$ (from Bit 4) at the corresponding positions. The coefficient *C* was found to be 0.0077 which indicates negligible interdependence between the two bitstreams, and thus the possibility of doubling the throughput capability of the RBG system.

We repeated the NIST SP 800-22 testing on data from Bit 3 for a large number of consecutive runs (200 runs, at 100 MS/s sampling rate, inter-electrode distance is $\sim$ 3–4 mm.). Each run consisted of switching ON the plasma system, acquiring 98 Mbits of data, and then switching it OFF. We found the success rates of 91.5%, 100%, 97.5%, 100%, 100%, 100%, 100%, 100%, 100%, 98.5%, 100%, 100%, 100%, 100% and 100%, for the 15 tests from Frequency to Linear Complexity, respectively. These results attest to the reliability of the APAMP system as a TRBG with no required binarization or post-processing treatment of the data. Lower than 100% success rates can be attributed mainly to the battery state-of-charge which lowers the overall power spectrum of the signal. For instance, when the battery (which is a 3.3 V, 4400 mA h rechargeable LIB) is fully charged, the percent success rates of 100 consecutive runs (100 Mbit in length collected directly from the ADC register at 100 MS/s) for passing all 15 tests were found to be 100%, 98%, 100%, $\ldots$, 100% for the test Frequency to Linear Complexity, respectively. Whereas when the battery is low in charge, the success rates were lower for the majority of tests: 65%, 70%, 68%, 70%, 70%, 100%, 70%, 100%, 70%, 70%, 70%, 100%, 100%, 70% and 100%, respectively.

## Discussion

Our goal with this study is to demonstrate the capability of APAMP system as a direct RBG based on its time-resolved current intensity dynamics with no post-processing. The source of these current fluctuations are most likely derived from the complex energy transfer processes occurring in the gas plasma, in addition to contributions from particles (positively- and negatively-charged, and neutral species) production/loss which result from the numerous possible chemical reactions^36^. These production/loss processes are nonlinear, collision-dominated, and take place with different kinetics and rate coefficients. Also, particles have different diffusion coefficients and mobilities in the gas medium, which makes the overall plasma state, and the resulting current signal in particular, very difficult to predict^32^. These fluctuations in current dynamics are usually linked to other fluctuations, such as pressure, plasma speed, and optical emissions^37^. It should also be noted that in practice other environmental and experimental sources of disturbances may add up coming from air flow turbulence, temperature noise, power supply ripples, electromagnetic radiation, etc.

Due to this inherent complexity in microplasma systems, a few theoretical attempts have been carried out to explain (at least conceptually) the origin of such fluctuations. For instance a notable work by Ghorui et al.^32,38^ (following the Arneodo et al. formalism^39^) demonstrated from basic governing equations, i.e. the conservation equations of mass, momentum, energy, and metal vapor concentration, together with Maxwell’s equation, that an amplitude equation^40^ describing the temporal evolution of perturbations of the plasma field quantities may be written as a third-order nonlinear differential equation of the form:

4 $$\begin{aligned} \dddot{A} + \mu _2 \ddot{A} + \mu _1 \dot{A} + \mu _0 A = k A^3 \end{aligned}$$

The coefficients $\mu _i$ are control parameters that depend on the properties of the generated plasma, and *k* is a scaling factor. Through a judicious choice of these parameters, this equation (also known as the jerk equation) shows that the general feature of the dynamic behavior of individual elements of plasma field vector may exhibit low-dimensional chaos. However, higher dimensional chaos (hyper-chaos) or more complex behavior cannot be explained by such a model. It is also understood that even if the general features can be somehow depicted by such a system of equations, which is qualitatively useful for the overall understanding of the system’s behavior, the exact one-to-one matching with the experiment is impossible to reproduce^41^. Based on the results presented in the previous section in which we showed the the suitability of bitstreams for RBG mainly from the NIST SP 800-22 point of view, these fluctuations cannot (retrospectively) be described by the set of equations 4. These equations are at the end initiated by deterministic processes and cannot pass directly all statistical tests of NIST SP 800-22. Further investigations on the physical origin of randomness in the APAMP system is beyond the scope of this work.

Now compared to other RBGs, the APAMP system we proposed here has several advantages. Today’s RBGs in Hardware Security Modules (HSMs), for instance, are circuits that rely on digital techniques such as harvesting phase noise in ring oscillators, or post-processing chaotic sequences generated by a chaotic oscillator. However, relying on these on-chip RBGs has its own limitations in terms of throughput, quality of random bits, and vulnerability to attacks which leads to securities issues and errors. For example, Differential Power Analysis (DPA) can be used to extract the data being processed by analyzing the current drawn by the processor from the supply. Defenses against this class of attacks by using, for example, random clocks or to randomly include no operation instructions (NOP) require some hardware overhead and increased design complexity. There are other physical means to generate random bits (but at lower speed) by harvesting noise from a number of sensors (e.g. temperature, humidity, visible light and infrared light sensors)^42^, or by timing the interval between two consecutive decays from a radioactive source^43^, and so on. These entropy sources have the advantage of being immune to power supply attacks, but also require subsequent digital signal post-processing to generate random bits. Our system, on the other hand, was proven to (i) directly provide truly random bit sequences from a physical source without the need of post-processing treatment, and (ii) be immune to external attacks given that the current fluctuation are at the high-voltage side of the transformer and the whole system is off-ship.

However, for high-speed real-time encryption applications, RBGs are mostly dependent on off-chip sources of entropy such as chaotic semiconductor lasers^25–28,44^, optical and non-optical quantum fluctuations^33,45,46^, and others^47^. Photonic devices with high bandwidth are the most popular options, and are able to reach ultra-fast bit rates of tens and hundreds of Gbit/s^27,48–50^ but at the cost of further post-processing routines that actually increase artificially the overall throughput (for example by means of higher-order derivatives^50^). The throughput of our APAMP system-based true RBG is lower than that, but its hardware simplicity and consequently low cost are much less. In addition, no data post-processing was required at any of the tested configurations. Nonetheless, improving the bit generation rate can be achieved by increasing the plasma generation power (may require higher capacity batteries), and/or combining more than one bit from the ADC module as we showed for Bit 3 and 4, and/or by using parallel acquisition lines for time-resolved current and optical emission intensities^5^.

## Conclusion

In this study we showed a very promising method and setup for generating high-rate, true random bits for RBG applications relying on the inherently stochastic behavior of current intensity in APAMP configuration. No post-processing routines are needed at any instance to obtain high-quality random bistreams that have been verified and validated using different packages of statistical tests. Besides, the complete system of microplasma generation and data acquisition can be made compact and inexpensive using off-the-shelf standard components, which makes it very competitive compared to the ones recently reported in the literature and commercially-available RBG modules.

## Supplementary information

Supplementary Information
