# Supplementary material for: Atmospheric pressure air microplasma current time series for true random bit generation
Source: Sci Rep. 2020 Dec 1;10:20971. doi: 10.1038/s41598-020-77956-5 (PMC7708637; doi:10.1038/s41598-020-77956-5)
Supplement: Supplementary file 1 — Supplementary Information [file 41598_2020_77956_MOESM1_ESM.pdf]

Atmospheric Pressure Air Microplasma Current Time Series for True Random Bit Generation

Anis Allagui[1,2,3,*], Sohaib Majzoub[4], Ahmed S. Elwakil[4,5,6], Andrea Espinel Rojas[2], and Hussain Alawadhi[7,2]

[1]Dept. of Sustainable and Renewable Energy Engineering, University of Sharjah, PO Box 27272, Sharjah, United Arab Emirates
[2]Research Institute of Sciences and Engineering, University of Sharjah, PO Box 27272, Sharjah, United Arab Emirates
[3]Dept. of Mechanical and Materials Engineering, Florida International University, Miami, FL33174, United States
[4]Dept. of Electrical Engineering, University of Sharjah, PO Box 27272, Sharjah, United Arab Emirates
[5]Nanoelectronics Integrated Systems Center, Nile University, Cairo 12588, Egypt
[6]Dept. of Electrical and Computer Engineering, University of Calgary, Calgary, Alberta T2N 1N4, Canada
[7]Dept. of Applied Physics and Astronomy, University of Sharjah, PO Box 27272, Sharjah, United Arab Emirates
[*]aallagui@sharjah.ac.ae

SUPPORTING INFORMATION

Experimental

Prototypes and setup

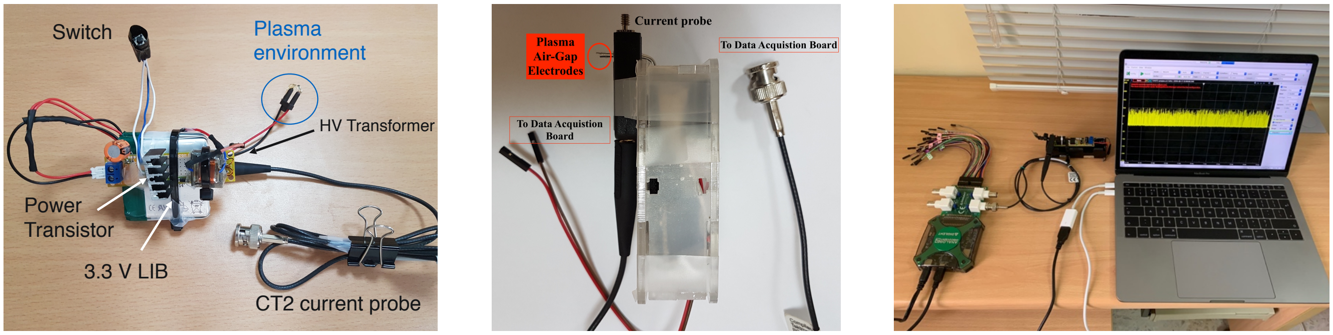

(a) (b) (c)

Figure S1. (a) Photograph of the battery-powered atmospheric pressure air microplasma prototype used for random bit generation in the manuscript. (b) Another prototype of the same circuit. (c) Photograph of the whole measurement setup including the Digilent Analog Discovery 2 (AD2) data acquisition board and computer; a sample of acquired current time series data appears on the computer screen.

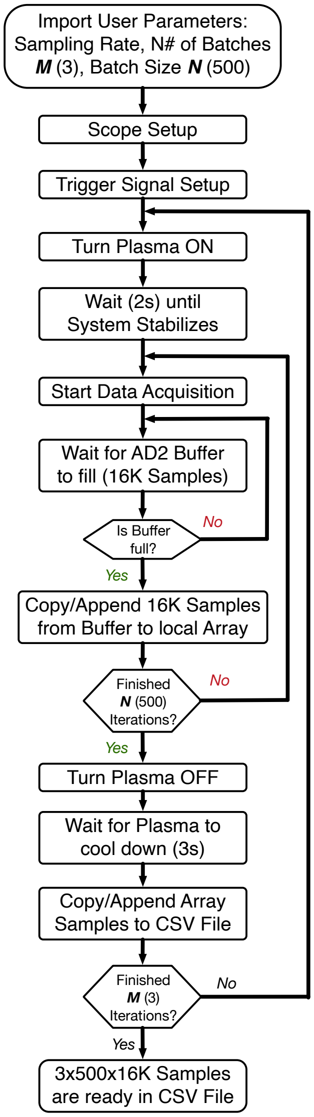

Figure S2. Flowchart depicting the steps used to collect the current time series from atmospheric pressure air microplasma system

Further results

Power spectra of current time series

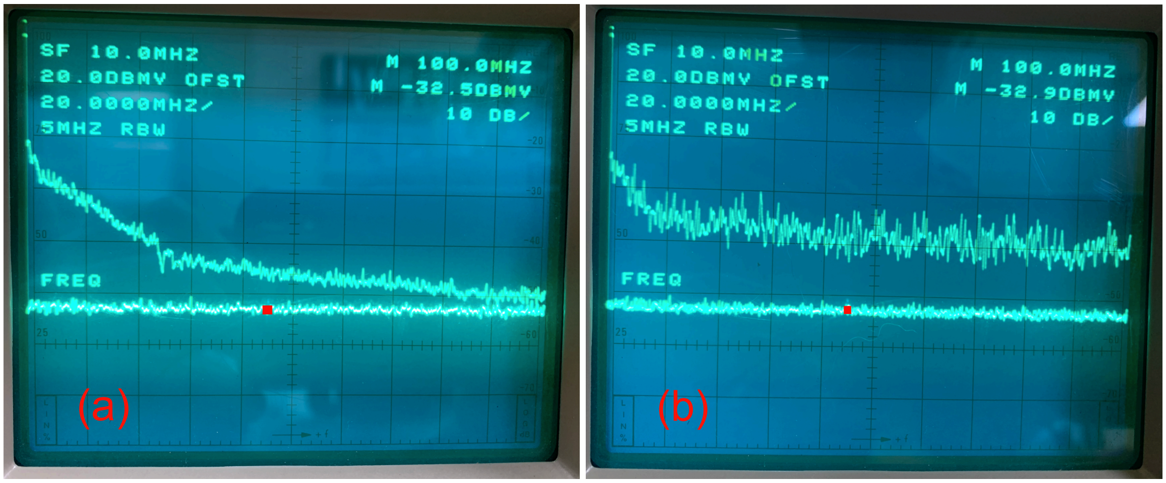

Figure S3. Power spectra of the generated current signals from atmospheric pressure air microplasma system compared to the noise floor (≈ -33 to -32 mdB) (a) when the two electrodes are close to each other and thus visual arcing is observed, and (b) when the two electrodes are spread apart and no visible arcing is observed. The frequency range spans 10 to 200 MHz and the central frequency of 100 MHz is indicated with a red marker.

Current time series when no arcing is observed

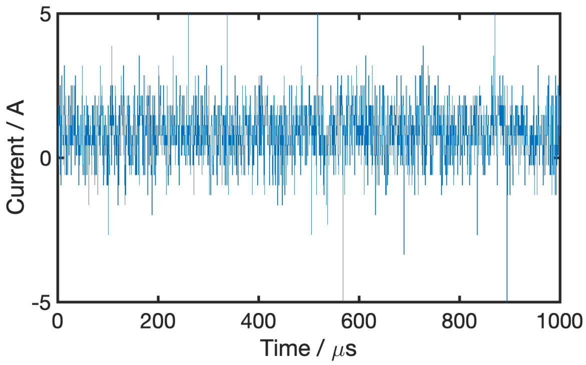

Figure S4. Current time series measured at 2 MS/s when the two electrodes are placed at a distance of ≈ 3-4 mm apart from each other. No plasma arc is visually observed under these conditions. The data show series of high-frequency, low-magnitude current signals compared to when the electrode are closer to each other (see Fig 2).

Additional NIST SP 800-22 tests results

Table S1. Typical NIST SP 800-22 tests results with $\alpha = 0.001$ performed on 100 Mbit-long bitstreams collected from Bits 3 of the ADC register at the sampling rate of 100 MS/s. The inter-electrode distance of the APAMP system is $\sim$ 3-4 mm (no visible current arc).

Statistical test	P-value	Proportion	Assessment
Frequency	0.739918	50/50	success
Block Frequency	0.383827	50/50	success
Cumulative Sums	0.350485	50/50	success
Runs	0.319084	50/50	success
Longest Run	0.455937	50/50	success
Rank	0.699313	50/50	success
FFT	0.494392	50/50	success
Non Overlapping Template	0.534146	50/50	success
Overlapping Template	0.574903	50/50	success
Universal	0.494392	50/50	success
Approximate Entropy	0.171867	50/50	success
Random Excursions	0.585209	23/23	success
Random Excursions Variant	0.484646	23/23	success
Serial	0.137282	49/50	success
Linear Complexity	0.494392	50/50	success

Dieharder (v. 3.31.1) tests results

Table S2. Typical Dieharder (v. 3.31.1) tests results performed on 100 Mbit-long bitstreams collected from Bit 3 of the ADC register at the sampling rate of 100 MS/s. The inter-electrode distance of the APAMP system is $\sim$ 3-4 mm (no visible current arc).

Statistical test	P-value	Assessment
Diehard Birthdays Test	0.98964959	passed
Diehard OPERM5 Test	0.12666850	passed
Diehard 32×32 Binary Rank Test	0.12803885	passed
Diehard 6×8 Binary Rank Test	0.23921223	passed
Diehard Bitstream Test	0.22100153	passed
Diehard OPSO	0.72002946	passed
Diehard OQSO Test	0.92026196	passed
Diehard DNA Test	0.09089890	passed
Diehard Count the 1s (stream) Test	0.71126845	passed
Diehard Count the 1s Test (byte)	0.02606059	passed
Diehard Parking Lot Test	0.82562887	passed
Diehard Minimum Distance (2d circle) Test	0.22940303	passed
Diehard 3d Sphere (minimum distance) Test	0.86994364	passed
Diehard Squeeze Test	0.03915401	passed
Diehard Sums Test	0.01417047	passed
Diehard Runs Test	0.61463327	passed
Diehard Craps Test	0.43741530	passed
Marsaglia and Tsang GCD Test	0.09290888	passed
STS Monobit Test	0.81832343	passed
STS Runs Test	0.30983887	passed
STS Serial Test (generalized)	0.65166684	passed
RGB Bit Distribution Test	0.50653467	passed
RGB Generalized Minimum Distance Test	0.34991903	passed
RGB Permutations Test	0.91478083	passed
RGB Lagged Sum Test	0.28657614	passed
RGB Kolmogorov-Smirnov Test	0.35138896	passed

* With acknowledgment to Dr Michail Maniatakos
(NYU Abu Dhabi, United Arab Emirates)

NIST SP 800-90B tests results

-10Mbin

IID Tests

Opening file: '10MbinArcing_a.bin'
Loaded 1000000 samples of 2 distinct 1-bit-wide symbols
Calculating baseline statistics...
    Raw Mean: 0.499921
    Median: 0.500000
    Binary: true

Literal MCV Estimate: mode = 500079, p-hat = 0.50007900000000005, p_u = 0.5013669152796566
H_original: 0.996061

Chi square independence
    score = 1955.327387
    degrees of freedom = 2046
    p-value = 0.923424

Chi square goodness of fit
    score = 2.414116
    degrees of freedom = 9
    p-value = 0.983105

**** Passed chi square tests**

LiteralLongest Repeated Substring results
    P_col: 0.5
    Length of LRS: 42
    Pr(X >= 1): 0.107454
**** Passed length of longest repeated substring test**

Beginning initial tests...

Initial test results
  excursion: 382.737
  numDirectionalRuns: 79533
  lenDirectionalRuns: 10
  numIncreasesDecreases: 74864
  numRunsMedian: 498659
  lenRunsMedian: 20
  avgCollision: 19.653
  maxCollision: 64
  periodicity(1): 24715
  periodicity(2): 24455
  periodicity(8): 24572
  periodicity(16): 24660
  periodicity(32): 24468
  covariance(1): 1.9996e+06
  covariance(2): 1.99919e+06
  covariance(8): 1.99884e+06
  covariance(16): 1.99871e+06
  covariance(32): 1.99787e+06
  compression: 155866

Beginning permutation tests... these may take some time
80.06% of Permutuation test rounds, 100.00% of Permutuation tests

statistic	C[i][0]	C[i][1]	C[i][2]
excursion	14	0	6
numDirectionalRuns	35	0	6
lenDirectionalRuns	9	5	1
numIncreasesDecreases	6	0	22
numRunsMedian	2001	0	6
lenRunsMedian	3	3	9
avgCollision	10	0	6
maxCollision	4	2	5
periodicity(1)	6	0	34
periodicity(2)	12	0	6
periodicity(8)	6	0	12
periodicity(16)	6	0	23
periodicity(32)	17	0	6
covariance(1)	6	0	6
covariance(2)	6	0	7
covariance(8)	30	0	6
covariance(16)	16	0	6
covariance(32)	88	0	6
compression	6	0	12

(* denotes failed test)

**** Passed IID permutation tests**

Restart tests

Opening file: '10MbinArcing_c.bin'
Loaded 1000000 samples made up of 2 distinct 1-bit-wide symbols.
H_I: 0.996061
ALPHA: 5.0251553006530614e-06, X_cutoff: 573
X_max: 552

Restart Sanity Check Passed...

Running IID tests...

Running Most Common Value Estimate...
Literal MCV Estimate: mode = 500196, p-hat = 0.50019599999999997, p_u = 0.50148391519677915
    Most Common Value Estimate (Rows) = 0.995725 / 1 bit(s)
Literal MCV Estimate: mode = 500196, p-hat = 0.50019599999999997, p_u = 0.50148391519677915
    Most Common Value Estimate (Cols) = 0.995725 / 1 bit(s)

H_r: 0.995725
H_c: 0.995725
H_I: 0.996061

Validation Test Passed...

min(H_r, H_c, H_I): 0.995725

Figure S5. Typical NIST SP 800-90B tests results performed on bitstreams generated from the microplasma current time series at 10 MS/s sampling rate for the case of visible arcing (inter-electrode distance of the APAMP system is $\sim$ 1 mm).

-50Mbin

IID Tests

Opening file: '50MbinArcing_a.bin'
Loaded 1000000 samples of 2 distinct 1-bit-wide symbols
Calculating baseline statistics...
    Raw Mean: 0.499367
    Median: 0.500000
    Binary: true

Literal MCV Estimate: mode = 500633, p-hat = 0.50063299999999999, p_u = 0.50192091426362484
H_original: 0.994468

Chi square independence
    score = 1922.406495
    degrees of freedom = 2046
    p-value = 0.975116

Chi square goodness of fit
    score = 19.706196
    degrees of freedom = 9
    p-value = 0.019815

**** Passed chi square tests**

LiteralLongest Repeated Substring results
    P_col: 0.500001
    Length of LRS: 38
    Pr(X >= 1): 0.837806
**** Passed length of longest repeated substring test**

Beginning initial tests...

Initial test results
  excursion: 757.905
  numDirectionalRuns: 79873
  lenDirectionalRuns: 10
  numIncreasesDecreases: 74704
  numRunsMedian: 499579
  lenRunsMedian: 19
  avgCollision: 19.8245
  maxCollision: 62
  periodicity(1): 24343
  periodicity(2): 24916
  periodicity(8): 24531
  periodicity(16): 24606
  periodicity(32): 24777
  covariance(1): 1.99456e+06
  covariance(2): 1.99566e+06
  covariance(8): 1.9958e+06
  covariance(16): 1.9948e+06
  covariance(32): 1.99601e+06
  compression: 155823

Beginning permutation tests... these may take some time
77.41% of Permutuation test rounds, 100.00% of Permutuation tests

statistic	C[i][0]	C[i][1]	C[i][2]
excursion	6	0	338
numDirectionalRuns	6	0	13
lenDirectionalRuns	11	6	0
numIncreasesDecreases	12	0	6
numRunsMedian	19	0	6
lenRunsMedian	4	4	2
avgCollision	6	0	19
maxCollision	28	3	3
periodicity(1)	67	0	6
periodicity(2)	6	0	894
periodicity(8)	6	0	8
periodicity(16)	6	0	8
periodicity(32)	6	0	94
covariance(1)	14	0	6
covariance(2)	6	0	24
covariance(8)	6	0	70
covariance(16)	6	0	10
covariance(32)	6	0	467
compression	38	0	6

(* denotes failed test)

**** Passed IID permutation tests**

Restart tests

Opening file: '50MbinArcing_c.bin'
Loaded 1000000 samples made up of 2 distinct 1-bit-wide symbols.
H_I: 0.994468
ALPHA: 5.0251553006530614e-06, X_cutoff: 573
X_max: 565

Restart Sanity Check Passed...

Running IID tests...

Running Most Common Value Estimate...
Literal MCV Estimate: mode = 500164, p-hat = 0.50016400000000005, p_u = 0.50145191522645272
    Most Common Value Estimate (Rows) = 0.995817 / 1 bit(s)
Literal MCV Estimate: mode = 500164, p-hat = 0.50016400000000005, p_u = 0.50145191522645272
    Most Common Value Estimate (Cols) = 0.995817 / 1 bit(s)

H_r: 0.995817
H_c: 0.995817
H_I: 0.994468

Validation Test Passed...

min(H_r, H_c, H_I): 0.994468

Figure S6. Typical NIST SP 800-90B tests results performed on bitstreams generated from the microplasma current time series at 50 MS/s sampling rate for the case of visible arcing (inter-electrode distance of the APAMP system is $\sim$ 1 mm).

-100Mbin

IID Tests

Opening file: '100MbinArcing_a.bin'
Loaded 1000000 samples of 2 distinct 1-bit-wide symbols
Calculating baseline statistics...
    Raw Mean: 0.499861
    Median: 0.500000
    Binary: true

Literal MCV Estimate: mode = 500139, p-hat = 0.500139, p_u = 0.50142691524596461
H_original: 0.995889

Chi square independence
    score = 2004.825635
    degrees of freedom = 2046
    p-value = 0.738131

Chi square goodness of fit
    score = 11.948117
    degrees of freedom = 9
    p-value = 0.216250

**** Passed chi square tests**

LiteralLongest Repeated Substring results
    P_col: 0.5
    Length of LRS: 44
    Pr(X >= 1): 0.0280193
**** Passed length of longest repeated substring test**

Beginning initial tests...

Initial test results
  excursion: 540.807
  numDirectionalRuns: 79731
  lenDirectionalRuns: 11
  numIncreasesDecreases: 74762
  numRunsMedian: 499097
  lenRunsMedian: 23
  avgCollision: 19.6985
  maxCollision: 69
  periodicity(1): 24548
  periodicity(2): 24496
  periodicity(8): 24845
  periodicity(16): 24755
  periodicity(32): 24528
  covariance(1): 1.99807e+06
  covariance(2): 1.99902e+06
  covariance(8): 1.99835e+06
  covariance(16): 1.99989e+06
  covariance(32): 1.99871e+06
  compression: 155898

Beginning permutation tests... these may take some time
75.77% of Permutuation test rounds, 100.00% of Permutuation tests

statistic	C[i][0]	C[i][1]	C[i][2]
excursion	6	0	28
numDirectionalRuns	6	0	13
lenDirectionalRuns	1	5	1
numIncreasesDecreases	7	0	6
numRunsMedian	99	0	6
lenRunsMedian	3	3	32
avgCollision	8	0	6
maxCollision	6	0	12
periodicity(1)	6	0	8
periodicity(2)	12	0	6
periodicity(8)	6	0	288
periodicity(16)	6	0	80
periodicity(32)	6	0	6
covariance(1)	26	0	6
covariance(2)	6	0	13
covariance(8)	15	0	6
covariance(16)	6	0	181
covariance(32)	6	0	20
compression	6	0	12

(* denotes failed test)

**** Passed IID permutation tests**

Restart tests

Opening file: '100MbinArcing_c.bin'
Loaded 1000000 samples made up of 2 distinct 1-bit-wide symbols.
H_I: 0.995889
ALPHA: 5.0251553006530614e-06, X_cutoff: 571
X_max: 567

Restart Sanity Check Passed...

Running IID tests...

Running Most Common Value Estimate...
Literal MCV Estimate: mode = 500487, p-hat = 0.50048700000000002, p_u = 0.50177491468482494
    Most Common Value Estimate (Rows) = 0.994888 / 1 bit(s)
Literal MCV Estimate: mode = 500487, p-hat = 0.50048700000000002, p_u = 0.50177491468482494
    Most Common Value Estimate (Cols) = 0.994888 / 1 bit(s)

H_r: 0.994888
H_c: 0.994888
H_I: 0.995889

Validation Test Passed...

min(H_r, H_c, H_I): 0.994888

Figure S7. Typical NIST SP 800-90B tests results performed on bitstreams generated from the microplasma current time series at 100 MS/s sampling rate for the case of visible arcing (inter-electrode distance of the APAMP system is $\sim$ 1 mm).

IID tests

Opening file: 'AD2CPP_Fri_P4_1_1M.bin'
Loaded 1000000 samples of 2 distinct 1-bit-wide symbols
Calculating baseline statistics...
Raw Mean: 0.500122
Median: 0.500000
Binary: true

Literal MCV Estimate: mode = 500122, p-hat = 0.50012199999999996, p_u = 0.50140991525739353
H_original: 0.995938

Chi square independence
score = 2008.360056
degrees of freedom = 2046
p-value = 0.719589

Chi square goodness of fit
score = 8.760145
degrees of freedom = 9
p-value = 0.459703

**** Passed chi square tests**

LiteralLongest Repeated Substring results
P_col: 0.5
Length of LRS: 36
Pr(X >= 1): 0.999308
**** Passed length of longest repeated substring test**

Beginning initial tests...

Initial test results
excursion: 327.143
numDirectionalRuns: 79771
lenDirectionalRuns: 10
numIncreasesDecreases: 74645
numRunsMedian: 499385
lenRunsMedian: 21
avgCollision: 19.9611
maxCollision: 66
periodicity(1): 24560
periodicity(2): 24610
periodicity(8): 24426
periodicity(16): 24490
periodicity(32): 24661
covariance(1): 2.00127e+06
covariance(2): 2.00158e+06
covariance(8): 2.00098e+06
covariance(16): 2.00127e+06
covariance(32): 2.00046e+06
compression: 155860

Beginning permutation tests... these may take some time
75.48% of Permutation test rounds, 100.00% of Permutation tests

statistic	C[i][0]	C[i][1]	C[i][2]
excursion	36	0	6
numDirectionalRuns	6	0	16
lenDirectionalRuns	7	6	0
numIncreasesDecreases	54	0	6
numRunsMedian	44	0	6
lenRunsMedian	3	3	14
avgCollision	6	0	179
maxCollision	5	2	4
periodicity(1)	8	0	6
periodicity(2)	9	0	6
periodicity(8)	37	1	5
periodicity(16)	7	0	6
periodicity(32)	6	0	15
covariance(1)	6	0	14
covariance(2)	6	0	18
covariance(8)	6	0	16
covariance(16)	6	0	32
covariance(32)	6	0	10
compression	6	0	7

(* denotes failed test)

**** Passed IID permutation tests**

Restart tests

Opening file: 'AD2CPP_Fri_P4_1_1M_b.bin'
Loaded 1000000 samples made up of 2 distinct 1-bit-wide symbols.
H_I: 0.995938
ALPHA: 5.0251553006530614e-06, X_cutoff: 572
X_max: 556

Restart Sanity Check Passed...

Running IID tests...

Running Most Common Value Estimate...
Literal MCV Estimate: mode = 500370, p-hat = 0.50036999999999998, p_u = 0.50165791494310097
Most Common Value Estimate (Rows) = 0.995224 / 1 bit(s)
Literal MCV Estimate: mode = 500370, p-hat = 0.50036999999999998, p_u = 0.50165791494310097
Most Common Value Estimate (Cols) = 0.995224 / 1 bit(s)

H_r: 0.995224
H_c: 0.995224
H_I: 0.995938

Validation Test Passed...

min(H_r, H_c, H_I): 0.995224

Figure S8. Typical NIST SP 800-90B tests results performed on bitstreams generated from the microplasma current time series at 100 MS/s sampling rate for the case of no visible arcing (inter-electrode distance of the APAMP system is ∼ 3-4 mm).

IID TESTS

Opening file: 'AD2CPP_Fri_P4_1_1M.bin'
Loaded 1000000 samples of 2 distinct 1-bit-wide symbols
Calculating baseline statistics...
Raw Mean: 0.500122
Median: 0.500000
Binary: true

Literal MCV Estimate: mode = 500122, p-hat = 0.50012199999999996, p_u = 0.50140991525739353
H_original: 0.995938

Chi square independence
score = 2008.360056
degrees of freedom = 2046
p-value = 0.719589

Chi square goodness of fit
score = 8.760145
degrees of freedom = 9
p-value = 0.459703

**** Passed chi square tests**

LiteralLongest Repeated Substring results
P_col: 0.5
Length of LRS: 36
Pr(X >= 1): 0.999308
**** Passed length of longest repeated substring test**

Beginning initial tests...

Initial test results
excursion: 327.143
numDirectionalRuns: 79771
lenDirectionalRuns: 10
numIncreasesDecreases: 74645
numRunsMedian: 499385
lenRunsMedian: 21
avgCollision: 19.9611
maxCollision: 66
periodicity(1): 24560
periodicity(2): 24610
periodicity(8): 24426
periodicity(16): 24490
periodicity(32): 24661
covariance(1): 2.00127e+06
covariance(2): 2.00158e+06
covariance(8): 2.00098e+06
covariance(16): 2.00127e+06
covariance(32): 2.00046e+06
compression: 155860

Beginning permutation tests... these may take some time
Permutation Test (core 0): 0 tests passed
Permutation Test (core 0): 1 tests passed
Permutation Test (core 0): 4 tests passed
Permutation Test (core 0): 7 tests passed
Permutation Test (core 0): 10 tests passed
Permutation Test (core 0): 11 tests passed
Permutation Test (core 0): 12 tests passed
Permutation Test (core 0): 14 tests passed
Permutation Test (core 0): 15 tests passed
Permutation Test (core 0): 16 tests passed
Permutation Test (core 0): 17 tests passed
Permutation Test (core 0): 18 tests passed

statistic	C[i][0]	C[i][1]	C[i][2]
excursion	26	0	6
numDirectionalRuns	6	0	10
lenDirectionalRuns	8	6	0
numIncreasesDecreases	48	0	6
numRunsMedian	101	0	6
lenRunsMedian	5	1	21
avgCollision	6	0	82
maxCollision	6	0	8
periodicity(1)	6	0	8
periodicity(2)	6	0	10
periodicity(8)	35	0	6
periodicity(16)	12	1	5
periodicity(32)	5	1	18
covariance(1)	6	0	21
covariance(2)	6	0	12
covariance(8)	6	0	10
covariance(16)	6	0	15
covariance(32)	6	0	12
compression	5	1	5

(* denotes failed test)

**** Passed IID permutation tests**

RESTART TESTS

Opening file: 'AD2CPP_Fri_P4_1_1M.bin'
Loaded 1000000 samples made up of 2 distinct 1-bit-wide symbols.
H_I: 0.995938
ALPHA: 5.0251553006530614e-06, X_cutoff: 571
X_max: 555

Restart Sanity Check Passed...

Running IID tests...

Running Most Common Value Estimate...
Literal MCV Estimate: mode = 500122, p-hat = 0.50012199999999996, p_u = 0.50140991525739353
Most Common Value Estimate (Rows) = 0.995938 / 1 bit(s)
Literal MCV Estimate: mode = 500122, p-hat = 0.50012199999999996, p_u = 0.50140991525739353
Most Common Value Estimate (Cols) = 0.995938 / 1 bit(s)

H_r: 0.995938
H_c: 0.995938
H_I: 0.995938

Validation Test Passed...

min(H_r, H_c, H_I): 0.995938

Figure S9. Typical NIST SP 800-90B tests results performed on bitstreams collected from Bits 3 of the ADC register at the sampling rate of 100 MS/s. The inter-electrode distance of the APAMP system is $\sim$ 3-4 mm (no visible current arc).
